# Hypoxia signaling controls postnatal changes in cardiac mitochondrial morphology and function

**DOI:** 10.1016/j.yjmcc.2014.06.013

**Published:** 2014-09

**Authors:** Marianne T. Neary, Keat-Eng Ng, Marthe H.R. Ludtmann, Andrew R. Hall, Izabela Piotrowska, Sang-Bing Ong, Derek J. Hausenloy, Timothy J. Mohun, Andrey Y. Abramov, Ross A. Breckenridge

**Affiliations:** a MRC National Institute for Medical Research, Mill Hill, London NW7 1AA; b UCL Institute of Neurology, Queen Square, London WC1N 3BG; c The Hatter Cardiovascular Institute, London WC1E 6HX; d UCL Division of Medicine, London WC1E 6JJ

**Keywords:** VHL, Von Hippel–Lindau factor, HIF, Hypoxia inducible factor, Pgc1, Peroxisome proliferator-activated receptor gamma co-activator 1, TMRM, Tetramethylrhodamine, methyl ester, MFN, Mitofusin, Cardiac, Neonatal, Hypoxia, Mitochondria, Mitofusin

## Abstract

Fetal cardiomyocyte adaptation to low levels of oxygen *in utero* is incompletely understood, and is of interest as hypoxia tolerance is lost after birth, leading to vulnerability of adult cardiomyocytes. It is known that cardiac mitochondrial morphology, number and function change significantly following birth, although the underlying molecular mechanisms and physiological stimuli are undefined.

Here we show that the decrease in cardiomyocyte HIF-signaling in cardiomyocytes immediately after birth acts as a physiological switch driving mitochondrial fusion and increased postnatal mitochondrial biogenesis. We also investigated mechanisms of ATP generation in embryonic cardiac mitochondria. We found that embryonic cardiac cardiomyocytes rely on both glycolysis and the tricarboxylic acid cycle to generate ATP, and that the balance between these two metabolic pathways in the heart is controlled around birth by the reduction in HIF signaling. We therefore propose that the increase in ambient oxygen encountered by the neonate at birth acts as a key physiological stimulus to cardiac mitochondrial adaptation.

## Highlights

• The reduction in HIF signaling encountered by the heart following birth acts as a physiological switch.
• Reduced postnatal cardiac HIF signaling affects mitochondrial number, structure and function.
• Experimental study of mitochondria is prone to artifacts due to the effect of oxygen.
• Cardiomyocytes employ multiple strategies to function in low oxygen *in utero.*

## 1 Introduction

Fetal cardiomyocytes are adapted to function in low levels of oxygen *in utero*, whereas adult cardiomyocytes are extremely vulnerable to hypoxia, as evidenced by high rates of mortality and morbidity caused by ischemic heart disease [1]. Shortly after birth, cardiomyocytes lose their capability to adapt to hypoxic conditions. The process underlying this postnatal maturational process is not understood.

It is thought that fetal cardiomyocytes generate ATP predominantly by glycolysis and glucose oxidation [2]. At birth, ambient oxygen levels increase sharply as the neonate emerges into the terrestrial environment. Immediately after birth, cardiac output, and therefore cardiac energy requirements, increase rapidly [3]. This increase is achieved through switching from glycolysis to β-oxidation of lipids, which generates larger amounts of ATP per unit of substrate than glycolysis [2,4], and is accompanied by an increase in mitochondrial number [5].

Recently, our understanding of molecular mechanisms controlling postnatal cardiac bioenergetics and mitochondrial remodeling has advanced, but physiological stimuli controlling these processes remain to be defined. This information is of interest with respect to future strategies to protect adult cardiomyocytes in ischemic heart disease and to increase our understanding of human cardiac conditions presenting around birth.

Mitochondrial biogenesis is upregulated following birth as a consequence of increases in *Pgc1α/β* expression [5], although the physiological stimuli for this are yet to be described. Furthermore, it is recognized that mitochondrial morphology is intimately linked with function, fusion of mitochondria being associated with increased β-oxidation of lipid [6,7]. Fetal mitochondria are “fragmented” in appearance, whereas postnatal mitochondria appear elongated, consistent with the metabolic switch occurring in the heart flowing birth [2]. Mitofusin proteins (MFNs) are implicated in the control of postnatal mitochondrial fusion, although, again, the relationship to physiological changes around birth is not known [8].

We investigated the mechanisms controlling neonatal cardiac mitochondrial remodeling at birth. Immediately following birth, ambient levels of oxygen encountered by the neonate increase significantly [9]. We have recently shown that the postnatal decrease in cardiac hypoxia signaling controls the lipid metabolizing gene expression program [10] and postnatal remodeling of the cardiac conduction system [11]. Additionally, cell culture experiments have shown that overexpression of hypoxia inducible factor (HIF) leads to diminished respiratory capacity and inhibited mitochondrial biogenesis [12,13]. We therefore hypothesized that the increase in cardiac oxygen tension acts, via HIF signaling, as a developmental “switch”, driving postnatal maturation of the heart. We investigated the effects of the reduction in hypoxia signaling following birth on the structure and function of cardiac mitochondria.

## 2 Results

### 2.1 The morphology of cardiac mitochondria around birth is dependent on hypoxia signaling

*In situ* measurement of mitochondrial function *in utero* is not currently possible without exposing the cells to oxygen, leading to artifacts due to oxygen-induced maturational processes. We therefore studied hearts from mice born into normobaric hypoxia (Fi0_2_ 10%) and *αMHC-Cre::VHL^(fl/fl)^* mice which maintain constitutively high levels of HIF signaling in the presence of normoxia [14] in order to inhibit postnatal maturational processes dependent on postnatal reductions in HIF signaling. We also generated stable HL1 cell lines transfected with an shRNA construct directed against VHL mRNA, which has a similar effect of stabilizing HIF expression in normoxia (Supplementary Fig. 1).

**Fig. 1:**
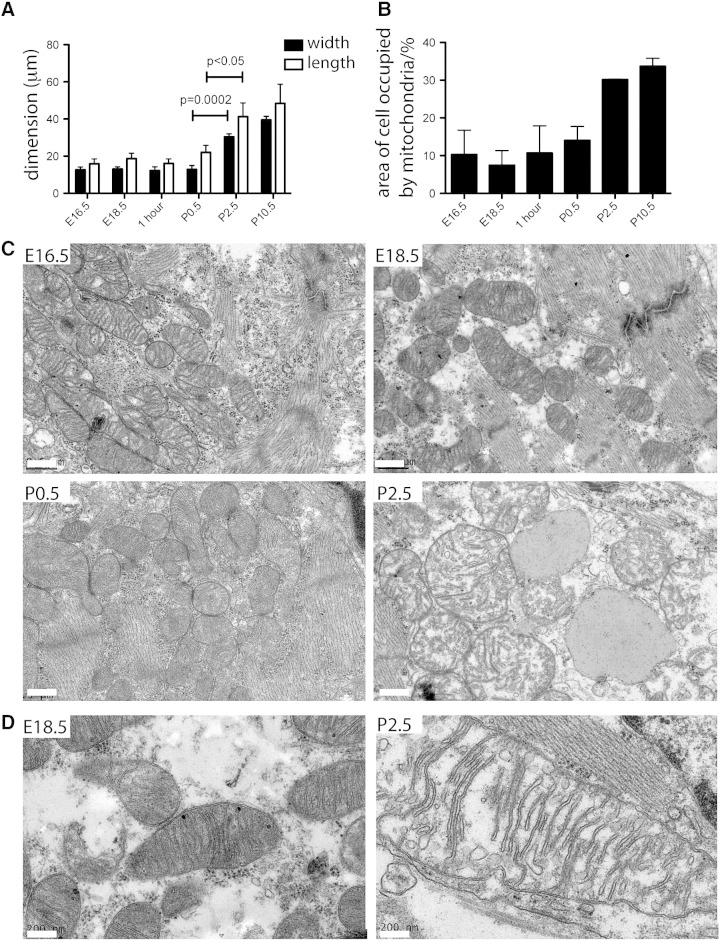
Mitochondrial size and number increases in cardiomyocytes after birth. (A) Average width and length of cardiac mitochondria increase as does % of the cell occupied by mitochondria (B) following birth, as measured from electron micrographs (n = 3 cells for each stage, p value calculated by 2-tailed t-test). Data presented as mean ± SEM. (C) Transmission electron micrographs, showing cardiomyocyte ultrastructure remodeling around birth. Between E18.5 and P2.5 inner mitochondrial membrane cristae become more dense and uniformly distributed within the mitochondria, and lipid droplets are visible in cardiomyocytes after the first day following birth (red asterisks in P2.5 image) Scale bars are 500 nm. (D) High power longitudinal view of mitochondria from E18.5 and P2.5 wild-type mouse hearts, showing dense mitochondrial matrix in E18.5 mitochondria-Scale bars 200 nm for these two images.

Perinatal changes in mitochondrial morphology have been observed in the mouse [15] and rabbit [16] from day 3 onwards and in the guinea pig [17] sheep [18], and mouse [8] around birth. These studies used electron microscopy, and consistently observed a shift in mitochondrial morphology from many small and ovoid to fewer long and rectangular mitochondria with an increase in proximity between mitochondria and the myofibrils with time after birth. In concordance with these previously published studies, we observed that mitochondria change shape from small and round before birth to larger and rectangular following birth (Fig. 1A and Supplementary Fig. 2). We estimated that percentage area occupied by mitochondria in the cell increases from E16.5 (10%) to P10.5 (34%), with the largest increase occurring in the first few days after birth between P0.5 and P2.5 (Fig. 1B). We observed that the mitochondrial cristae become more dense and uniform between E16.5 and P10.5, with an observable decrease in glycogen storage granules sequentially from E16.5 to P10.5 and lipid droplets observed on micrographs from the day of birth onwards (P0.5) (Fig. 1C and Supplementary Fig. 2). We noted that mitochondrial morphology changes during the perinatal period. Mitochondrial matrix in P2.5 mitochondria appears less dense, and sarcomeres more dense and defined on EMs compared with E18.5 mitochondria (Fig. 1D and Supplementary Fig. 2).

**Fig. 2:**
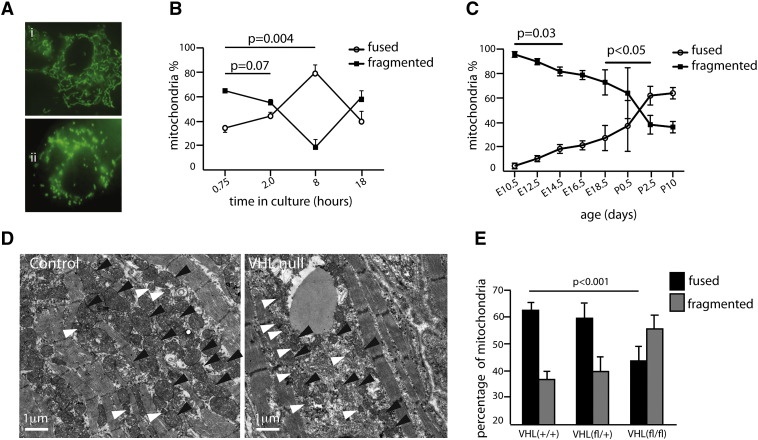
Decreased postnatal in cardiac HIF signaling leads to an increased proportion of fused cardiomyocyte mitochondria. (A) Fluorescence micrograph of primary cultured E16.5 cardiomyocytes stained with MitoTracker showing fused (i) and fragmented (ii) mitochondrial morphology. (B) Quantification of mitochondrial morphology in primary cultures of cardiomyocytes at varying times after isolation and 45 min of MitoTracker staining, n = 3 each group, scored by observer blinded to the experiment. P value represents 2-tailed t test. The proportion of fused mitochondria (open circles) increases with gestational age, and rapidly increases following birth. (C) Representative electron micrographs of cardiomyocytes from control *αMHC-Cre* and *αMHC-Cre::VHL^(fl/fl)^* hearts (VHL Null), showing smaller mitochondria with fewer cristae in VHL null cells. (D) Representative electron micrographs of left ventricular myocyte from control *αMHC-Cre* and *αMHC-Cre::VHL^(fl/fl)^* P2.5 hearts, showing a decreased mitochondrial number and increased proportion of fragmented mitochondria in the VHL null cardiomyocyte compared with control (black arrowheads towards fused mitochondria, and white arrowheads towards fragmented mitochondria). (E) The proportion of fused and fragmented mitochondria is reversed in αMHC-Cre::VHL^(fl/fl)^, with more fragmented than fused mitochondria visible in VHL null P10 cardiomyocytes (n = 9, 7 and 9 in the VHL *^(+/+)^,*VHL *^(fl/+)^ and* VHL *^(fl/fl)^* groups, respectively, p value represents 2-tailed t test).

We went on to examine whether perinatal changes in cardiac mitochondrial morphology are dependent on changes in ambient oxygen levels and hypoxia signaling immediately following birth. We found that interpreting mitochondrial morphology in electron micrographs was difficult, due to plane of sectioning issues. Therefore, we used primary cultures of cardiomyocytes, which enable the use of vital dyes to assess mitochondrial morphology more sensitively than electron microscopy (Fig. 2A). Primary culture of embryonic cardiac cells involves removal from a hypoxic environment *in vivo* and exposure to potentially artifactually high levels of oxygen in culture. We therefore assessed the effect of cardiomyocyte culture time on fusion phenotype of the heart cells. We digested and plated E18.5 embryonic hearts and found that the length of time in culture increased the proportion of fused mitochondria until 8 h post-plating (p = 0.004) (Fig. 2B). At a culture time of 18 h the association reversed. This emphasizes the difficulty in interpretation of mitochondrial morphology after prolonged periods of cell culture. Cardiomyocyte viability was not compromised by 45-min incubation with MitoTracker dye: at 24 h after MitoTracker treatment, we observed adherent cells, often displaying synchronized beating.

Observers blinded to their origin examined primary cell cultures of cardiomyocytes, after 45-min of MitoTracker dye treatment in cells one hour after plating. We found an increase in the proportion of cardiac cells exhibiting mainly fused mitochondria from E18.5 to P10.5. We observed an increase in the numbers of fused mitochondria after birth with only 27% mitochondria being fused at E18.5 compared to 62% fused at P2.5 (p = 0.05) (Fig. 2C).

At birth, the mammalian embryo encounters a sharp increase in ambient oxygenation [9]. We have previously shown that cardiac levels of HIF1α protein, a key mediator of hypoxia signaling fall, but are elevated in neonatal hearts homozygous null for VHL [10]. We therefore investigated whether neonatal changes in cardiac HIF signaling drive the changes in cardiac morphology, number and function in the neonatal heart.

To assess the impact of hypoxia signaling on mitochondrial morphology we examined electron micrographs of hearts from postnatal day 10.5 (P10.5) *αMHC-Cre::VHL^(fl/fl)^* mice. Mitochondria from *αMHC-Cre::VHL^(fl/fl)^* mice appeared immature with respect to controls (Fig. 2D). The image in Fig. 2D is taken from a αMHC-Cre heart (constitutive cre expressing), which is on a C57BL/10 background as opposed to the (CBA/Ca × C57BL/10) strain used for the wild-type analysis outlined in Fig. 1 and Supplementary Fig. 2. This strain difference could explain the different mitochondrial morphology observed between these two images. Further examples of mitochondrial morphology are pictured in Supplementary Fig. 2. In primary cultures of cardiomyocytes using Mitotracker dye, we found that mitochondria from P10 *αMHC-Cre::VHL^(fl/fl)^* hearts exhibited significantly higher numbers of fragmented-appearing mitochondria compared with controls (p < 0.001) (Fig. 2E).

### 2.2 Expression of mitochondrial fusion proteins is controlled in the heart by hypoxia signaling

**Fig. 3:**
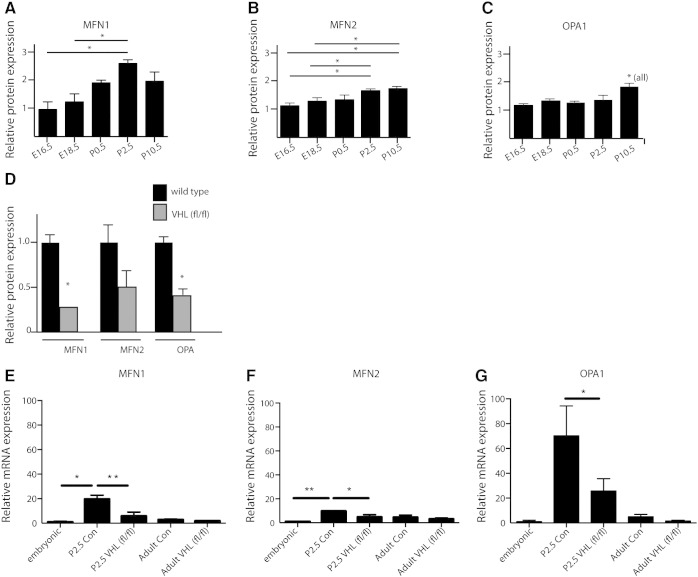
Cardiac expression of MFN1, MFN2 and OPA1 in the neonatal heart is affected by HIF signaling. (A–C) Quantification of western blot of protein extract from wild-type mouse hearts at E16.5–P10.5, showing significant increases in postnatal cardiac protein levels of MFN1, MFN2, OPA1 respectively (n = 3 each experiment, *P < 0.05, p value represents 2-tailed t test). (D) Postnatal cardiac levels of MFN1, MFN2 and OPA1 are significantly reduced in *αMHC-Cre::*VHL *^fl/fl^* hearts compared with αMHC-Cre controls (n = 6, *P < 0.05, p value represents 2-tailed t test). Data presented as mean ± SEM. (E–G) Quantitative RT-PCR of cDNA from embryonic (E18.5), postnatal (P2.5)αMHC-Cre (control) and αMHC-Cre::VHL^(fl/fl)^ hearts, and adult mercremer::VHL^(fl/fl)^ following 5 days of tamoxifen induction, showing postnatal transcriptional upregulation of MFN1, MFN2 and OPA1 at birth in all cases; this rise is significantly attenuated in P2.5 VHL null hearts, which do not downregulate HIF signaling postnatally. Cardiac expression of MFN1, MFN2 and OPA1 mRNA is not altered following cardiac-specific deletion of VHL. Adult mercremer::VHL^(fl/fl)^ mice were given tamoxifen IP for 5 days at 30 days of age, and analyzed 30 days after the first injection. (n = 6, *P < 0.05, **P < 0.005, p value represents 2-tailed t test). Data presented as mean ± SEM.

Mitochondrial morphology is regulated by a family of mitochondrial fusion and fission proteins [8,19]. We therefore investigated expression of the fusion proteins, *MFN1*, *MFN2* and *OPA1*, and fission proteins, *DRP1* and *FIS1* at regular intervals from E16.5 to P10.5. Western blotting of protein extracts of ventricular tissue revealed an increase in fusion protein expression from E16.5 in a stepwise manner through to P10.5 with the largest increase taking place between P0.5 and P2.5 in *MFN1* and *MFN2* (p = 0.05) and between P2.5 and P10.5 in *OPA1* (p = 0.05) (Figs. 3A–C, representative blots in Supplementary Fig. 3). There were no changes detectable in fission protein *DRP1* and *FIS1* levels over this time period (Supplementary Fig. 4). We found that in *αMHC-Cre::VHL^(fl/fl)^* hearts, expression of fusion proteins *MFN1* and *OPA1* was significantly reduced compared with controls (P < 0.001 and p = 0.04 respectively, 2 tailed t tests n = 4 hearts each group) (Fig. 3D). We analyzed cardiac mRNA expression of MFN1, MFN2 and OPA1. We found that levels of all three mRNAs increase significantly in the heart at birth, and this rise is significantly attenuated in hearts from *αMHC-Cre::VHL^(fl/fl)^* neonates (Figs. 3E–G). These data suggest that postnatal HIF-dependent mitochondrial fusion could be mediated by increases in *MFN1, MFN2* and *OPA1* expression. Interestingly, no significant change in mRNA levels of MFN1, MFN2 and OPA1 was seen in adult inducible, cardiac specific VHL deleting mercremer::*VHL^(fl/fl)^* hearts, following 5 days of tamoxifen treatment.

**Fig. 4:**
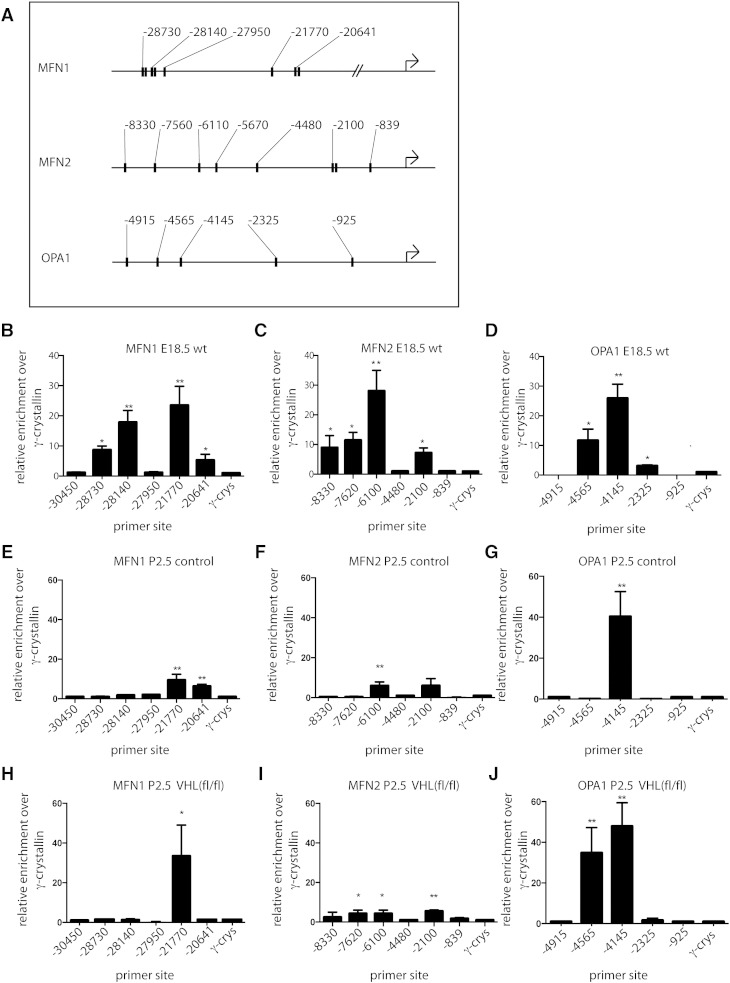
Binding of HIF1α to the 5’ promoters of mitofusins is affected by hypoxia signaling in the perinatal heart. (A) Schematic of 5’ promoters of MFN1, MFN2 and OPA1 showing locations of canonical (N)CGTG HIF1α binding sites. (B–D) RTPCR of chromatin immunoprecipitation assay using anti HIF1α antiserum and primers to the HIF motif-containing sequences in MFN1, MFN2 and OPA1 promoters from e18 hearts from αMHC-Cre control hearts, showing binding of HIF1α to more than one site in each promoter. (E–G) HIF1α chromatin immunoprecipitation from control P2.5 cardiac chromatin revealed reduction in HIF1α binding to several sites in all promoters, and that binding was altered in chromatin from αMHC-Cre::VHL^(fl/fl)^ hearts (H–J). (Bars represent summation of three experiments, and results expressed as multiples of signal for non-amplified sequence. P values are 2-tailed t tests relative to non-amplified *γ-crystallin* primers. *p < 0.5, **p < 0.005).

We went on to examine whether HIF1α binds to the 5’ promoters of MFN1, MFN2 and OPA1. We analyzed published 5’ promoter sequences for these 5 genes, and found several canonical (A/G)CGTG HIF1α binding sites in each case (Fig. 4A). We assayed whether HIF1α directly binds these promoters using anti HIF1α serum to perform chromatin immunoprecipitation. We found that in each case, chromatin containing at least one HIF1α-binding site promoter fragment was enriched 20 fold over non-amplified sequences in chromatin from embryonic αMHC-Cre control hearts (Figs. 4B-D), implying that HIF1α binds to these sites *in vivo*. Chromatin Immunoprecipitation with anti-HIF1α serum in chromatin from P2.5 control hearts revealed differences in binding of HIF1α to several sites in these three promoters (Figs. 4E-F). These changes were not seen in their entirety in chromatin from P2.5 αMHC-Cre::VHL^(fl/fl)^ hearts (Fig. 4H–I). We performed immunoprecipitation for each promoter site in e18 cardiac chromatin using an isotype control IgG. We found no significant specific binding of this antibody to any promoter site. This implies that chromatin remodeling occurs in the heart after birth, and that, to a degree, this remodeling may be affected by HIF signaling.

### 2.3 Postnatal mitochondrial biogenesis is dependent on changes in oxygen concentration and HIF signaling

**Fig. 5:**
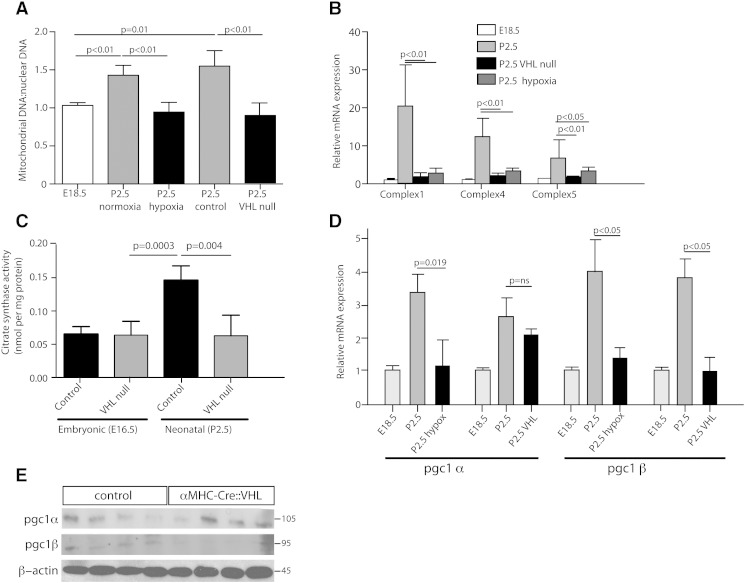
Hypoxia signaling controls postnatal cardiomyocyte mitochondrial biogenesis. (A) PCR of mitochondrial-encoded (*COX2*) and nuclear-encoded (*β-globin*) genes revealed an increase in mitochondrial DNA at P2.5 compared with E18. This increase was not seen in *αMHC-Cre::VHL^(fl/fl)^* hearts (VHL null), or hearts from animals born into 10% oxygen (hypoxia) (n = 6 hearts each group, p value represents 2-tailed t test). (B) RTPCR of cardiac cDNA isolated from neonatal hearts revealed that postnatal increases in transcription of mitochondrial-encoded subunits of Complex 1, 4 and 5 are not seen in *αMHC-Cre::VHL^(fl/fl)^* hearts, or hearts from wild-type mice born into 10% FiO_2_. (C) Cardiac citrate synthase activity, a marker of mitochondrial volume, increases at birth in control hearts, but this increase is not seen in *αMHC-Cre::VHL^(fl/fl)^* hearts. (N = 8 each group, p = two tailed t test). (D) RTPCR showing *Pgc1α/β* mRNA levels increase in the heart after birth in control but not hearts from mice born into 10% oxygen (hypoxia). mRNA levels of *Pgc1β* but not *Pgc1α* were significantly reduced in *αMHC-Cre::VHL^(fl/fl)^* hearts compared with wild types. (n = 6 hearts each group p value represents 2-tailed t test for all RTPCR experiments). (E) Western blots of protein extract from P2.5 *αMHC-Cre::VHL^(fl/fl)^* and control hearts, showing that levels of *Pgc1α* in *αMHC-Cre::VHL^(fl/fl)^* hearts do not differ from controls, whereas *Pgc1β* protein levels are lower in *αMHC-Cre::VHL^(fl/fl)^* hearts than controls.

We went on to investigate whether the previously observed increase in cardiac mitochondrial mass after birth is also dependent on HIF-signaling. We examined the mitochondrial: nuclear DNA ratio from DNA extracted from cardiac ventricular tissue at E18.5 (shortly before birth) and P2.5. We found a significant increase in the mitochondrial: nuclear DNA ratio between E18.5 and P2.5 in wild-type mouse hearts (Fig. 5A) (p < 0.01). This increase was not seen in hearts from *αMHC-Cre::VHL^(fl/fl)^* neonates or from mice born in 10% ambient oxygen, implying that postnatal cardiac mitochondrial biogenesis is dependent on downregulation of HIF signaling (Fig. 3A). We found that increased postnatal expression of several mitochondrial-encoded mRNAs is also inhibited in *αMHC-Cre::VHL^(fl/fl)^* hearts and hearts from P2.5 mice born and reared in 10% oxygen (hypoxia) (Fig. 5B).

We also examined myocardial citrate synthase activity in *αMHC-Cre::VHL^(fl/fl)^* and control hearts. Citrate synthase activity has been shown to reflect the overall volume of mitochondria present in a sample, a proxy for mitochondrial number [20]. We found that citrate synthase activity increases 2.5 fold between E16.5 and P2.5 (p = 0.0003, n = 6 each group) in wild type mouse hearts consistent with our finding of an increased proportion of cardiomyocytes occupied by mitochondria and increased mitochondrial: nuclear DNA ratio postnatally (Fig. 5C). Examination of hearts from *αMHC-Cre::VHL^(fl/fl)^* P2.5 mice revealed no postnatal increase in citrate synthase activity, implying that a drop in postnatal HIF signaling is required for increased mitochondrial biogenesis.

The *Pgc1α/β* proteins have been shown to control neonatal mitochondrial biogenesis [5]. We therefore examined whether postnatal changes in HIF signaling and/or ambient oxygen levels lead to altered expression of *Pgc1α/β.* We found a postnatal increase in *Pgc1*α mRNA expression following birth in control hearts, as previously reported [5]; we found no significant difference in *Pgc1*α mRNA expression between P2.5 *αMHC-Cre::VHL^(fl/fl)^* and control hearts (Fig. 5D). However, we observed a significant decrease in *Pgc1*α RNA expression in cardiac ventricles from mice born in 10% ambient oxygen compared with normoxic controls (p = 0.019, 2 tailed t-test, 4 hearts each group). *Pgc1β* mRNA expression was decreased in ventricles from both *αMHC-Cre::VHL^(fl/fl)^* and wild-type hypoxic mice compared with controls (Fig. 5D). Western blotting of protein extracts from ventricles of E16.5 and P2.5 *αMHC-Cre::VHL^(fl/fl)^* and control mouse hearts revealed that the pattern of *Pgc1α/β* protein expression broadly mirrors that of mRNA in these hearts (Fig. 5E). This implies that *Pgc1α* expression in the heart around birth is not dependent on HIF signaling, whereas *Pgc1β* expression is.

Taken together, our data suggest that postnatal increases in mitochondrial mass and changes in mitochondrial morphology are driven by increased ambient oxygen levels and decreased HIF signaling immediately after birth. The exact molecular mechanisms underlying this hypoxia-dependent mitochondrial biogenesis remain unclear.

### 2.4 HIF signaling controls neonatal cardiac mitochondrial function

We went on to investigate the effects of high levels of HIF signaling on mitochondrial function in the embryonic heart. It is well known that increases in *HIF1α* expression in a wide variety of cell types induce metabolic changes that lead to lower oxygen consumption [21]. We therefore examined mitochondrial function and oxygen consumption in primary cultures from *αMHC-Cre::VHL^(fl/fl)^* and control hearts, as well as HL1 cardiomyocyte cell lines stably transfected with an shRNA construct directed against VHL.

**Fig. 6:**
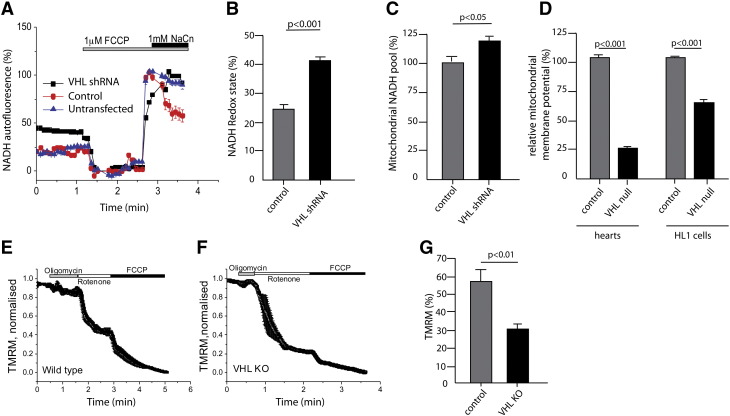
HIF signaling controls postnatal changes in mitochondrial redox state. (A) NADH autofluorescence in empty-vector-transfected control and ShRNA *VHL* HL-1 cells in response to FCCP (1 μM) and NaCN (1 mM). Data have been normalized and expressed as percentage of maximum. (B) NADH redox state (%) and (C) mitochondrial NADH pool (%) in control and shRNA *VHL* cells. Data are presented as mean of 3 experiments compared to controls. (D) Basal mitochondrial membrane potential in control and primary cardiomyocytes from *VHL* null hearts or *VHL* knockdown HL1 cell lines. Data presented as mean percentage compared to WT ± SEM. (E, F) TMRM fluorescence in WT and *VHL* KO primary cardiomyocytes respectively after addition of oligomycin (2 μg/ml), rotenone (1 μM) and FCCP (1 μM). (G) TMRM fluorescence in control (WT) and *VHL* null primary cultured cardiomyocytes in response to FCCP after application of oligomycin 2 μg/ml and rotenone 1 μg/ml. Data presented as mean percentage compared to control ± SEM.

Application of the mitochondrial uncoupler FCCP (1 μM) to cells maximizes respiration and completely oxidizes the mitochondrial pool of NADH, manifesting as a decrease in fluorescence (**taken as 0**) (Fig. 5A). Conversely, the complex IV inhibitor NaCN (1 mM) suppresses respiration by preventing NADH oxidation and allowing the NADH pool to regenerate fully (**taken as 100**). The redox index is expressed as a percentage of basal NADH autofluorescence between 0 and 100 [22]. Cardiomyocytes isolated from E16.5 *αMHC-Cre::VHL^(fl/fl)^* hearts exhibited an increase in mitochondrial NADH redox index when compared to controls (Figs. 6A–B) suggesting an inhibition of mitochondrial respiration. Interestingly, the rate of regeneration of the NADH pool in response to NaCN addition was much slower in VHL knockdown cells than the corresponding rate in control cells (Fig. 6A) suggesting a mild inhibition of NADH production in the tricarboxylic acid cycle (TCA). Despite the slower NADH production the total mitochondrial pool of NADH (estimated as a difference in signal between application FCCP and NaCN) was significantly higher than control cells (Fig. 6C). This suggests that the inhibition of mitochondrial respiration in VHL null cells (i.e. with constitutively active HIF signaling) is not due to lack of substrate for complex I.

Mitochondrial respiration is dependent on the mitochondrial membrane potential (Δψ_m_). Using live cell imaging and the fluorescent dye tetramethylrhodamine methyl ester (TMRM), we measured the Δψ_m_, a universal indicator of mitochondrial health and the metabolic state of the cell. We observed a dramatic decrease in the level of Δψ_m_ in VHL-knockdown HL1 cells (to 60% actual of control, n = 14 cells per genotype) and even lower in *αMHC-Cre::VHL^(fl/fl)^* cardiomyocytes (to 25% of control, n = 12 cells; Fig. 6D). In healthy cells, Δψ_m_ is maintained by the activity of the mitochondrial respiratory chain. In the event of damage or an inhibition of mitochondrial respiration, cells may maintain Δψ_m_ using ATP hydrolysis through the ATP synthase [23]. In order to investigate the mechanism of maintenance of Δψ_m_ in hypoxic cardiomyocytes, mitochondrial inhibitors were applied and their effects on Δψ_m_ were observed. Application of the F_0_-F_1_-ATP synthase inhibitor oligomycin (2 μg/ml) did not significantly change Δψ_m_, while subsequent inhibition of complex I by rotenone (1 μM) caused a rapid loss of potential (Fig. 6E). Despite the low Δψ_m_, VHL knockdown cells did not depolarize in response to oligomycin but showed a small hyperpolarization, suggesting that the mitochondria produce ATP in conditions of a low Δψ_m_ (Fig. 6F). Subsequent application of rotenone and FCCP led to a drop in the signal in response to the inhibitor of complex I that was greater in VHL knockdown cells when compared to control cells. In VHL knockdown cells, a lower response to FCCP at the end of the experiment suggests that complex II plays a smaller part in the maintenance of Δψ_m_ as previous application of oligomycin and rotenone leaves complex II as the only electron donor under these conditions (Fig. 6G).

**Fig. 7:**
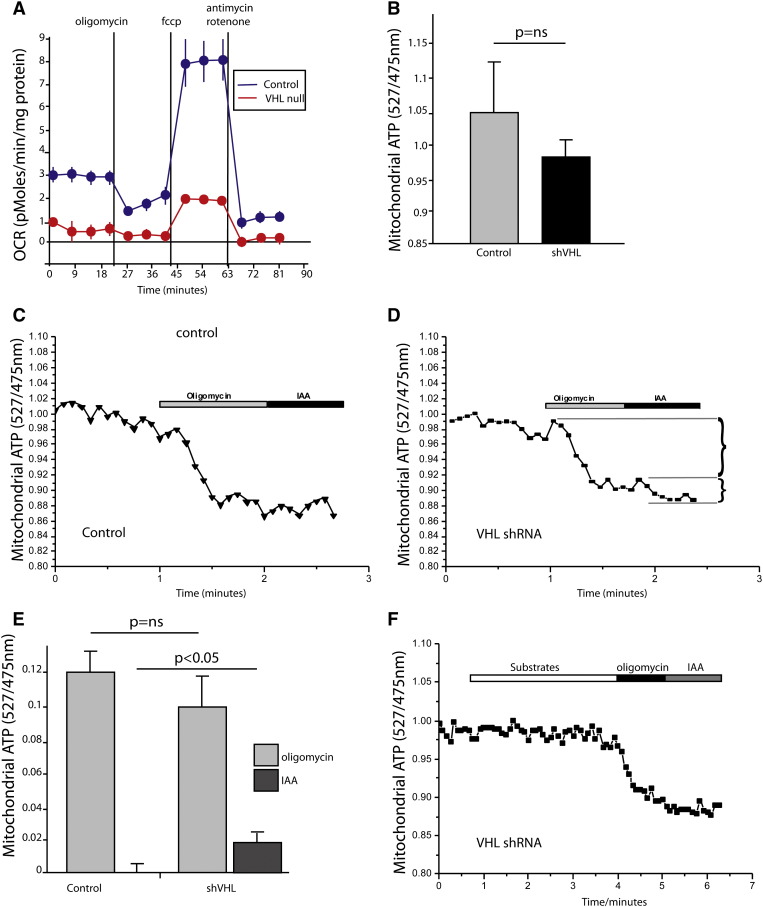
Hypoxic cardiac mitochondria generate ATP via glycolysis and the TCA cycle. (A) Mitochondrial respiration profiles (OCR = oxygen consumption rate) of control and *VHL* null primary cardiomyocytes were obtained using the Seahorse XF Cell Mito Stress Test Kit. Data are represented as mean ± S.E.M. of 5 biological replicates. (B) Quantification of basal ATP levels in control and *VHL* knockdown HL1 cells (shVHL). (C–D) Live cell measurements of ATP levels in control and shVHL HL1 cells in response to oligomycin (2 μg/ml) and iodoacetic acid (20 μM). (E) Quantification of the decrease in ATP levels upon oligomycin and iodoacetic acid exposure as indicated in (D). (F) Live cell measurement of ATP in shVHL HL1 cells after the addition of mitochondrial substrates (5 mM glutamate, 5 mM malate, 5 mM succinate and 1 mM palmitoyl carnitine) and exposure to oligomycin as well as iodoacetic acid. Data presented as mean ± SEM.

Measurement of cell respiration using a SeahorseXF analyzer showed lower levels of oxygen consumption in *αMHC-Cre::VHL^(fl/fl)^* cardiomyocytes (Fig. 7A). The inhibitory effect of oligomycin (which reflects respiration coupled to ATP production) was also significantly lower in VHL null cells in comparison to controls cells implying lower levels of oxidative phosphorylation (n = 5 assays for each genotype, experiment repeated 3 times). This difference was also reflected in the maximal rate of oxygen consumption (measured after stimulation with the uncoupler FCCP), which was also significantly reduced in *αMHC-Cre::VHL^(fl/fl)^* cardiomyocytes (Fig. 7A), implying that downregulation of cardiac HIF signaling is necessary for postnatal mitochondrial remodeling. We performed oxygen flux analysis on VHL knockdown and control HL1 cells and found a similar result (Supplementary Fig. 5).

The reduced respiration and its insensitivity to oligomycin, lower mitochondrial membrane potential in VHL null cardiomyocytes indicate that oxidative phosphorylation could be impaired in these cells. We therefore compared the ATP levels in control and VHL knockdown HL1 cells with the ATP sensing probe Cox AT1.03 [24,25]. Surprisingly, we found no significant difference in mitochondrial ATP production between control (empty vector) and VHL knockdown HL1 cells (Fig. 7B). We also investigated the impact on ATP production of inhibiting oxidative phosphorylation (by inhibition with oligomycin, 2 μg/ml) or glycolysis (by inhibition with iodoacetic acid (IAA), 20 μM). Compared to empty-vector transfected controls, the basal level of ATP in VHL knockdown cells showed no significant difference (n = 6) (Figs. 7C, D). Most importantly, the use of inhibitors revealed that the mechanism of ATP production differs between VHL knockdown and control cells. As expected, in control cells oligomycin depleted all ATP and IAA had no further effect (Fig. 7C). In VHL-knockdown cells, oligomycin induced a decrease in ATP levels but it also significantly induced a drop in ATP in response to the inhibitor of glycolysis IAA (Figs. 7D-E), implying that VHL-knockdown cells rely not only on oxidative phosphorylation but also heavily on glycolysis for their ATP production. Interestingly, pre-incubation of VHL-knockdown cells with substrates for mitochondrial respiration (5 mM glutamate, 5 mM malate, 5 mM succinate and 1 mM palmitine–carnitine) did not change the level of [ATP] but completely blocked the effect of IAA on [ATP] (Fig. 7F). Thus, a small proportion of the glycolysis to ATP production in VHL-knockdown cells can be inhibited by recovery of oxidative phosphorylation by addition of mitochondrial substrates. We suggest that provision of several substrates stimulates oxidative phosphorylation and reduces the need for glycolysis in hypoxic cardiomyocytes.

## 3 Discussion

Here we describe data supporting a novel hypothesis that the postnatal increase in oxygen tension encountered by the neonate acts as a physiological trigger for mitochondrial maturation in the heart. We found that mitochondrial morphology changes dramatically around birth, with mitochondria assuming an elongated form postnatally. These changes in mitochondrial morphology are dependent on a reduction in cardiac HIF signaling, and that HIF-dependent changes in expression of *MFN1* and *OPA1* may mediate this. The postnatal increase in cardiac mitochondrial biogenesis is also dependent on a decrease in HIF signaling. Postnatal downregulation of HIF signaling also leads to increased respiratory capacity and oxidative phosphorylation. This work has implications for medical conditions such as cyanotic congenital heart disease, where tissues are inadequately oxygenated following birth due to abnormal cardiac anatomy. This work focuses on cardiac mitochondrial changes around birth; it is not yet known how these relate to mechanisms regulating adult cardiac mitochondrial function and morphology.

We found no evidence that the postnatal increase in transcription of *Pgc1*α is dependent on a decrease in HIF-signaling, in agreement with our earlier finding that upregulating of *HIF1α* in HL1 cells does not lead to an increase in *Pgc1α* protein levels [10]. Our finding that the rise in *Pgc1α* mRNA and Pgc1α protein levels is attenuated in mice born into low levels of ambient oxygen agrees with the finding that *Pgc1α* expression is hypoxia dependent, but HIF-independent in mouse skeletal muscle [26]. Interestingly, the changes seen in the heart at birth are the reverse of skeletal hypoxic adult skeletal muscle—i.e. hypoxia *in utero* inhibits expression of *Pgc1α*. This does leave the question open as to what does transcriptionally regulate *Pgc1α* in the heart around birth. Certainly increases in expression of *Pgc1α* are coincident with those of *MFN1* and *MFN2* in the heart around birth, and the phenotypes of *Pgc1α/β* and *MFN1/2* null mice have similarities [5,8]. However, our findings that postnatal increased *Pgc1α* transcription is hypoxia- but not HIF-dependent, suggest parallel regulatory pathways. Our finding that postnatal levels of pgc1β in the mouse heart are reduced in VHL null hearts supports the finding that HIF negatively regulates pgc1β expression in renal carcinoma cells [27].

Immediately following birth, in addition to changes in ambient oxygen/HIF signaling, the ambient temperature of the neonate drops, and levels of several hormones and signaling pathways change, although these changes remain poorly defined. Many of these changes have been identified as stimuli to *Pgc1α* expression in the context of “stress” [28], and are thus candidates for regulatory triggers for neonatal cardiac maturation. We are still some way away from a complete understanding of the physiological regulation of neonatal cardiac remodeling. It is unclear whether the HIF system is maximally activated at 10% FiO_2_. We chose this oxygen concentration as it is the lowest level before significant mortality makes analysis impossible. Death rates of wide type neonatal mice at 10% FiO_2_ are still significantly higher than cardiac VHL null pups, however, and it is unclear whether any observed cardiac effects in these mice are cardiac-specific rather than due to the global effects of hypoxia.

Our understanding of mitochondrial function in the heart lags behind that of mitochondria in cell-types such as neuron, possibly because of the difficulty in culturing and studying cardiomyocytes. We believe that study of the changes occurring in the heart around birth will afford an insight into the endogenous physiological mechanisms adapting the embryonic heart to extremely low levels of oxygen *in utero.* This information may be of use in devising novel strategies to protect vulnerable adult cardiomyocytes, for example during myocardial infarction.

The changes in, and regulation of gene expression in the heart around birth are not fully understood. Our chromatin immunoprecipitation data suggest that there could be significant remodeling of cardiac chromatin immediately following birth, and that this may be HIF dependent. Oxygen-dependent chromatin modification has been described [29]. The role of chromatin modification versus direct HIF1α promoter binding in the transcriptional control of Mitofusin expression is as yet unclear, and will be necessary to define completely the control of neonatal Mitofusin expression by promoter analysis. Analysis of genomic binding sites of HIF1α led to the conclusion that HIF1α acts as a transcriptional activator for the vast majority of putative targets [30]. However, some examples of HIF1α acting as a repressor have been published [10,27,31]. Our data suggest that HIF1α is acting as a transcriptional repressor in the context of neonatal cardiac Mitofusin expression, although formal proof of this is needed. We found no difference in HIF1α protein levels in E18.5 VHL null and control hearts, implying that the HIF system is maximally active in the fetal heart (Supplementary Fig. 6). This, however, remains to be investigated formally. Furthermore, it should be noted that deletion of the VHL gene has HIF-independent effects on gene expression [32]. This is therefore a potential limitation of our study.

Our data suggest that the predominant mitochondrial site of adaptation to hypoxia is electron transport complex 1. Complex 1 has been found to be a target for HIF signaling in cardiac cells [33,34]. Chronic exposure of rats to low ambient levels of oxygen leads to adaptive changes in ROS generation along with decreases in activity of mitochondrial complexes I, II and IV. The relatively low density of mitochondria in embryonic cardiomyocytes is arguably an adaptation to low levels of oxygen, leading to lower ROS generation. This does raise the possibility that therapies directed towards inhibiting cardiac mitochondrial function may be beneficial in ischemic adult myocardium. Indeed, it could be argued that the field of ischemia protection via preconditioning and/or opening of the mitochondrial permeability transition pore (MPTP) is exactly this—inhibiting mitochondrial function in a similar manner to the embryonic cardiomyocyte.

We found that ATP is generated in VHL-null cardiomyocytes predominantly by oxidative phosphorylation with a small contribution of glycolysis. Importantly, despite the inhibition of mitochondrial respiration in hypoxic cardiomyocytes, oxidative phosphorylation is not completely inhibited, and we hypothesize that hypoxic cardiomyocytes are able to utilize a number of mitochondrial substrates allowing levels of oxidative phosphorylation sufficient to maintain ATP production. Our work outlining a novel mechanism whereby VHL-knockdown HL1 cells generate ATP and maintain an inner mitochondrial membrane gradient on a background of constitutive hypoxia signaling provides a mechanistic framework for future studies investigating how fetal cardiomyocytes are adapted to extreme hypoxia, and potential novel therapeutic approaches to cardioprotection. We would predict that treatments aimed at boosting mitochondrial ATP production rather than saving oxygen might benefit ischemic cells in some circumstances.

Finally, our data together with those of others, suggest that the embryonic myocardium is adapted to function in low levels of uterine oxygen by a number of parallel mechanisms. We have shown that postnatal expression of the genes encoding lipid-metabolizing genes is upregulated postnatally by an HIF-dependent mechanism [10]. Mitochondrial number is reduced in embryonic myocardium relative to neonatal, and activity of mitochondrial complex 1 and 4 is inhibited in the embryonic heart and hypoxic cardiac tissue. This suggests that a combination of several therapeutic strategies may be necessary to protect ischemic adult myocardium adequately.

## 4 Materials and methods

### 4.1 Animal husbandry

All mouse experiments were carried out in compliance with institutional ethical and welfare standards in accordance with Home Office Animal Procedures Act (1986). The transgenic mouse strain, αMHC-Cre::VHL^(fl/fl)^ on a C57BL/10 background is previously described [11]. PCR amplification was performed on tail-derived genomic DNA to determine genotype. αMHC-Cre mouse hearts were used as controls in this study, unless otherwise stated. Tamoxifen was administered to mercremer::VHL^(fl/fl)^ and mercremer control mice intraperitoneally dissolved in vegetable oil at a dose of 1 mg for 5 consecutive days starting at 30 days old, and were analyzed 30 days after the first tamoxifen injection.

### 4.2 Electron microscopy of mitochondria

Hearts were isolated from E10.5–E18.5 embryos from timed-mated pregnant mice, and from newborn mice, between 0.5 and 10.5 days old. Paired tail samples were collected for PCR genotyping. Left ventricular sections were prepared for electron microscopy as described previously and imaged using a Jeol 1200EX Transmission Electron Microscope with a Gatan Orius 1000 CCD.

Mitochondrial density was calculated by the number of mitochondria in a given area (128 μm^2^) at 2000 × magnification. The percentage area occupied by mitochondria was estimated by the density multiplied by the average length and width of the mitochondria, divided by the image area. An average over ten pictures was taken for each heart and three hearts were used per time point. Pictures were taken by serial panning and the user was blinded to the sample type.

### 4.3 Live mitochondrial imaging

Hearts were isolated from E10.5–E18.5 embryos from timed-mated pregnant mice, and from new born mice, between 0.5 and 10 days old. Paired tail samples were collected for PCR genotyping. Hearts were individually placed in a labeled tube containing 300 μl of warm 1 mg/ml Collagenase II solution (Worthington Chemicals), which was then transferred to a shaking incubator (37 °C) for 10 min. Collagenase was then removed and the hearts washed three times in warm Claycomb medium (Sigma-Aldrich). MitoTracker green (Life technologies), a green-fluorescent mitochondrial stain, was prepared to a 1 μM DMSO solution as per the manufacturer's specifications, and added to tissue culture medium at a concentration of 0.2 μl/ml. 500 μl of medium was transferred to the hearts in sterile tubes and a p1000 Pasteur pipette was used to disrupt the tissue and disaggregate the cells until there were no visible clumps of cells. The cells were plated onto a glass-bottomed petri dish (Mattek Corp) coated with 1% gelatin (Difco) and the volume of medium was made up to 2 ml total. The plate was incubated at 5% CO_2_ and 37 °C for 45 min after which the medium was washed and replaced with phenol-red free Dulbecco’s Modified Eagle’s Medium (DMEM). A Deltavision RT system based on an inverted Olympus IX70 microscope (Applied Precision Inc.), inside a heated chamber (Solent) at 37 °C, was used to capture single cell images using the Fluorescein IsothioCyanate (FITC) excitation and emitter filters and a 100**×** oil objective lens. MitoTracker Green signal is not sensitive to changes of mitochondrial conditions including membrane potential and redox status. Between 20 and 30 cells were imaged per heart sample. Images were randomized, anonymized, and then scored blindly and independently by two experts in mitochondrial morphology as either predominantly fragmented or fused. The scores were averaged.

### 4.4 Western blotting

Hearts from embryos and neonatal mice were dissected in cold PBS.

Hearts were homogenized in homogenization buffer (10% weight: volume) (PBS supplemented with protease and phosphatase inhibitors, Thermo Scientific). Samples were boiled and denatured in loading buffer containing 5% 2-mercaptoethanol (Invitrogen) and run on a 10% SDS PAGE gel. Samples were transferred to PVDF membrane and probed with antibodies for MFN1 (Abcam, UK, cat. no. ab57602), MFN2 (Abcam, UK, ab124773) OPA1 (Abcam, UK, ab90857) and DRP1 (Santa Cruz, USA cat. no. sc-271583). Blots were developed using ECL and hyperfilm (GE Healthcare, UK) and scanned before analysis using Image J.

### 4.5 Chromatin immunoprecipitation

This was carried out as previously described [10], using 4 μg rabbit anti-mouse HIF1α antibody (NB100-134, Novus Biochem). We performed immunoprecipitation for each promoter site in e18 cardiac chromatin using an isotype control IgG (Abcam ab13347000. We found no significant specific binding of this antibody to any promoter site.

### 4.6 Neonates raised in hypoxia versus normoxia

Pregnant F1 (CBA/Ca x C57BL/10) females were sacrificed at 18 days post coitus. The embryos were removed and fostered onto a Parkes mouse, which had littered the previous day. Neonates were raised by a foster mother for 24 h in either normoxia or in a hypoxic chamber (Custom Glovebox, Biospherix) maintained at 10% O_2_. Prior to this, foster mothers were acclimatized to increasing hypoxia for at least three days preceding the experiment, whereas the dam carrying the experimental neonates was maintained in normoxia until she was sacrificed and embryos removed. This eliminated maternal and prenatal exposure to hypoxia which may have affected postnatal phenotypes.

### 4.7 Citrate synthase activity assay

Hearts were snap frozen, and after PCR genotyping homogenized and subjected to colorimetric assay for citrate synthase (Biovision, California, USA) as per manufacturer’s instructions. Lowry protein assay was performed on the cardiac homogenate (BioRad). Results were normalized to protein content.

### 4.8 Gene expression studies

Immediately after dissection in cold PBS, hearts were snap frozen in liquid nitrogen and stored at − 80 °C. RNA was extracted from hearts using Trizol reagent (Invitrogen) according to the manufacturer's instructions. CDNA was prepared from RNA samples using Superscript III and random primers (Invitrogen). Quantitative RT-PCR was carried out using an ABI Prism 7000 cycler (Applied Bio systems) with the cDNA equivalent to 30 ng of total RNA per reaction using SYBR green (Thermo Scientific), with 18 s mRNA as the normalizing control. All PCR primers were purchased from Qiagen. Primers used Complex I; NDUF8, Complex 4 mtCOX2, Complex 5 ATP5a [35].

### 4.9 Embryonic cardiomyocyte isolation

Embryonic cardiac myocytes were isolated from E16–18 embryos from timed-pregnant mice. The mother was sacrificed by cervical dislocation and embryos were taken and stored at room temperature in freshly prepared ADS buffer (116 mM NaCl, 5.4 mM KCL, 20 mM HEPES, 0.8 mM NaH_2_PO_4_, 405.7 μM MgSO_4_, 5.5 mM glucose, pH 7.35). Hearts from each embryo were excised and rinsed in ADS buffer before they were stored in labeled 1.5 ml Eppendorf tubes. Tails samples were also obtained for PCR analysis. Tissue culture dishes (6 well plates or Seahorse plates) were coated with 1% gelatin (Difco) during the isolation steps.

The following steps were carried out under tissue culture conditions: Hearts were minced and serially digested in 1 ml digestion buffer (ABS buffer) containing 1 mg/ml Collagenase II (Worthington Chemicals) and 0.5 mg/ml Pancreatin (Sigma-Aldrich) in a shaking incubator set at 37 °C for 15 min. Myocytes were not removed from the first digestion therefore the supernatant from the first digest was discarded without removing the hearts. Fresh digestion buffer was added to each heart sample and left to incubate at 37 °C for 10 min. After this incubation, the digest was removed and transferred to 15 ml Falcon tubes containing 2 ml Fetal Calf serum (FCS) to stop the enzymic reaction. The supernatant collected from two digests were centrifuged at 1000 r.p.m for 3 min, the solution was discarded and the cell pellet was resuspended in fresh FCS. These incubation and centrifugation steps were repeated six to eight times until the cardiac myocytes were completely removed and the heart tissue was reduced to a single matrix conglomerate.

The final cell pellet from each heart was resuspended in Dulbecco’s Modified Eagle’s Medium (DMEM) containing 10% FCS and penicillin (50 U/ml), streptomycin (50 μg/ml) (Invitrogen). Fibroblasts were allowed to attach for 30 min and the non-adherent (myocyte) fraction was plated into either 6 well dishes containing 22 mm glass coverslips (VWR) for mitochondrial function analysis or Seahorse plates for oxygen consumption analysis. After 24 h, the myocytes formed a monolayer of spontaneously beating cells; the medium was removed and replaced with DMEM containing 1% FCS and 1% P/S before further analysis. Hearts from Cre positive or VHL floxed embryos were used as controls to compare against mutant VHL hearts.

### 4.10 ShRNA VHL stable cell lines

HL-1 cells were transfected with shRNA construct directed against VHL mRNA using FugeneHD (Invitrogen) according to manufacturer's instructions. 48 h after transfection, Claycomb medium was supplemented with 0.4 mg/ml G148 in order to select clones overexpressing shRNA VHL. Individual clones were picked and qRT-PCR and Western Blot verified expression levels of VHL. For oxygen consumption rate and mitochondrial function experiments, neomycin was removed several passages before analysis. An HL1 line stably expressing an empty vector was used as control in these studies, unless otherwise stated.

### 4.11 Oxygen consumption

A Seahorse Bioscience Instrument was used to measure oxygen consumption rate as per manufacturer's instructions [36]. For a detailed protocol, please refer to Breckenridge et al. 2013 [10]. All readings were normalized to protein content, by performing a Lowry protein content assay on wells following completion of the Seahorse assay.

### 4.12 Mitochondrial redox state

The redox state of mitochondrial NADH is a function of respiratory chain activity and substrate turnover. We measured the resting level of NADH autofluorescence in the cells, which was then expressed as the ‘redox index’, a ratio of the maximally oxidized and maximally reduced signals [37]. The dynamic range of the signals was defined by obtaining the maximally oxidized signal following the response to 1 μM FCCP (which stimulates maximal respiration and fully oxidizes the mitochondrial NADH pool). In these conditions, mitochondrial NADH is taken as fully oxidized and defined as 0%. The maximally reduced signal was then defined as the response to 1 mM NaCN (which fully inhibits respiration), preventing NADH oxidation, and so promoting maximal mitochondrial NADH reduction. In these conditions NADH is taken as 100% reduced. NADH autofluorescence measurements were obtained from ShRNA-VHL cells.

### 4.13 Measurement of mitochondrial redox state

NADH autofluorescence was measured using an epifluorescence inverted microscope equipped with a 20 × fluorite objective. Excitation light at a wavelength of 350 nm was provided by a Xenon arc lamp, the beam passing through a monochromator (Cairn Research, Kent, UK). Emitted fluorescence light was reflected through a 455 nm long-pass filter to a cooled CCD camera (Retiga, QImaging, Canada) and digitized to 12 bit resolution. Imaging data were collected and analyzed using software from Andor (Belfast, UK).

### 4.14 Measurement of mitochondrial membrane potential

Mitochondrial membrane potential (Δψ_m_) is an indicator of mitochondrial state. In these experiments tetramethylrhodamine methylester (TMRM) is used in the ‘redistribution mode’ to assess Δψ_m_, and therefore a reduction in mitochondrial localized TMRM fluorescence represents mitochondrial depolarization. We measured Δψ_m_, in shRNA VHL knockdown cell lines and in primary cultures. For measurements of ΔΨ_m_, cells were plated on 22 mm glass coverslips and loaded for 40 min at room temperature with 25 nM TMR (Invitrogen) in a HEPES buffered saline solution (HBSS) composed of 156 mM NaCl, 3 mM KCl, 2 mM MgSO_4_, 1.25 mM KH_2_PO_4_, 2 mM CaCl_2_, 10 mM glucose and 10 mM HEPES; pH adjusted to 7.35 with NaOH. The dye remained present in the media at the time of recording and is used in the redistribution mode. Therefore a reduction in TMRM fluorescence represents Δψ_m_ depolarization. Confocal images were obtained using a Zeiss 710 VIS CLSM equipped with a META detection system and a 40 × oil immersion objective. TMRM was excited using the 560 nm laser line and fluorescence was measured above 580 nm. For basal ΔΨ_m_ measurements, Z-stack images were obtained by confocal microscopy and analyzed using Zeiss software [22]. For measurements of ATP, the cells were transfected for 24 h with the ATP sensing probe Cox AT1.03 using Lipofectamine 2000 (Qiagen) according to manufacturer’s instructions [24,25].

### 4.15 Fluorescence measurements

NADH autofluorescence was determined with excitation at 351 nm and emission at 375–470 nm [22]. Measurements of ATP levels with Cox AT1.03 were obtained using measurement of fluorescence resonance energy transfer by exciting the cyan fluorescent protein at 405 nm (measured at 460 to 510 nm), used to excite yellow fluorescent protein, measured from 515 to 580 nm [24]. All data presented were obtained from at least 5 coverslips and 2–3 different cell preparations.

### 4.16 Statistical analysis

Statistical analysis was performed with Origin 8 (Microcal Software Inc., Northampton, MA, USA) software. Student’s t-test was applied. Means expressed ± the standard error of the mean (SEM).

## Financial statement

This work was part-funded by the British Heart Foundation, Project grant PG/10/51/28444 to R. B.

T.M. is funded by the Medical Research Council (U117562103).
