# Supplementary material for: Hypoxia signaling controls postnatal changes in cardiac mitochondrial morphology and function
Source: J Mol Cell Cardiol. 2014 Sep;74(100):340–52. doi: 10.1016/j.yjmcc.2014.06.013 (PMC4121533; doi:10.1016/j.yjmcc.2014.06.013)
Supplement: Supplementary file 1 — Supplementary material [file mmc1.docx]

**
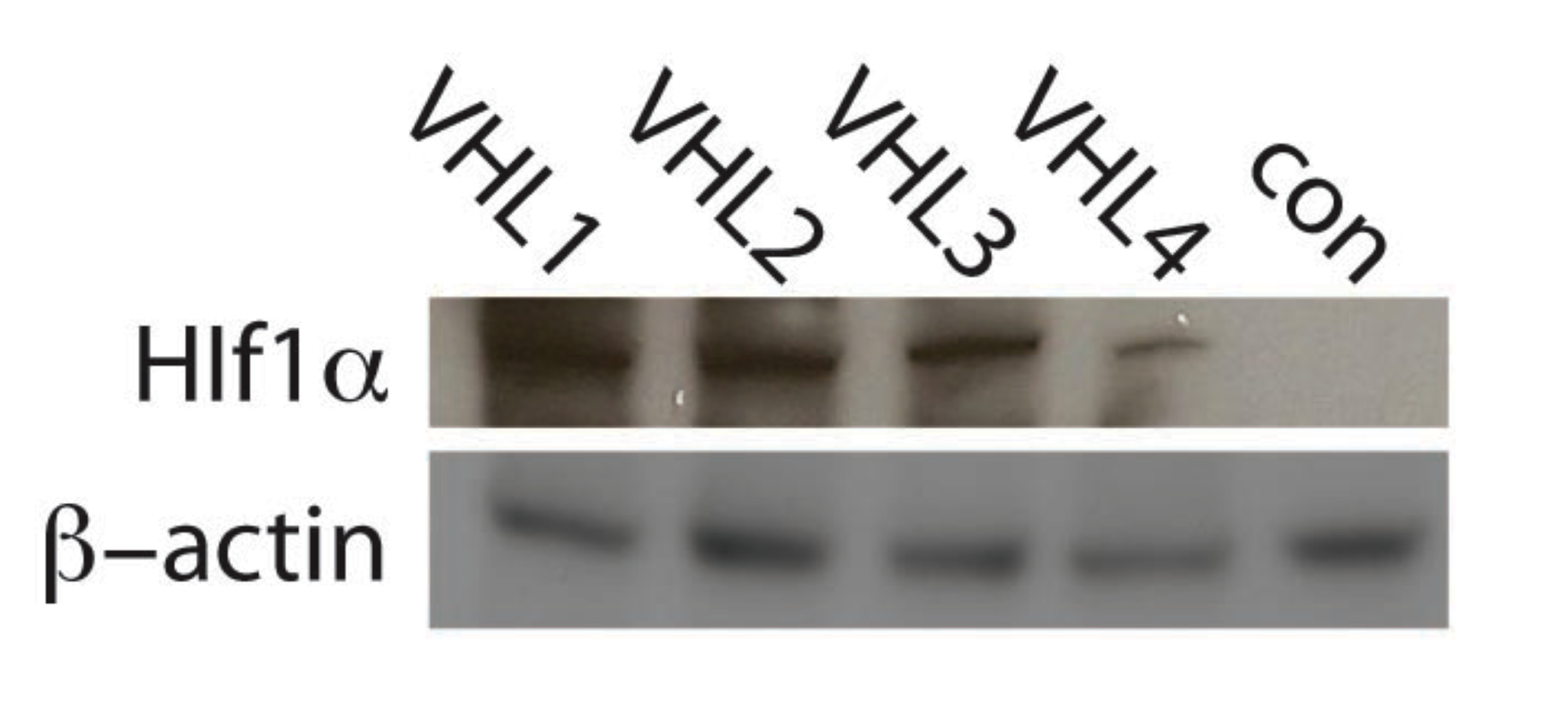
**

**Supplementary Figure 1**

HL1 cells transfected with a construct generating an shRNA directed against VHL lead to elevated HIF expression. Western blot showing HIF protein expression in 4 independent shRNA transfected lines, and a control line transfected with an empty vector. Line1 used throughout the studies unless stated otherwise.

**Supplementary figure 2. Perinatal maturation of mitochondrial cristae.**

Transmission electron micrographs show the maturation of the cristae. Between E18.5 and P10.5 inner mitochondrial membrane cristae become more densely and more uniformly distributed within the mitochondria. There was a reduction in glycogen granules (arrow) and just after birth, at P0.5, many lipid droplets were visible (labeled ‘L’).


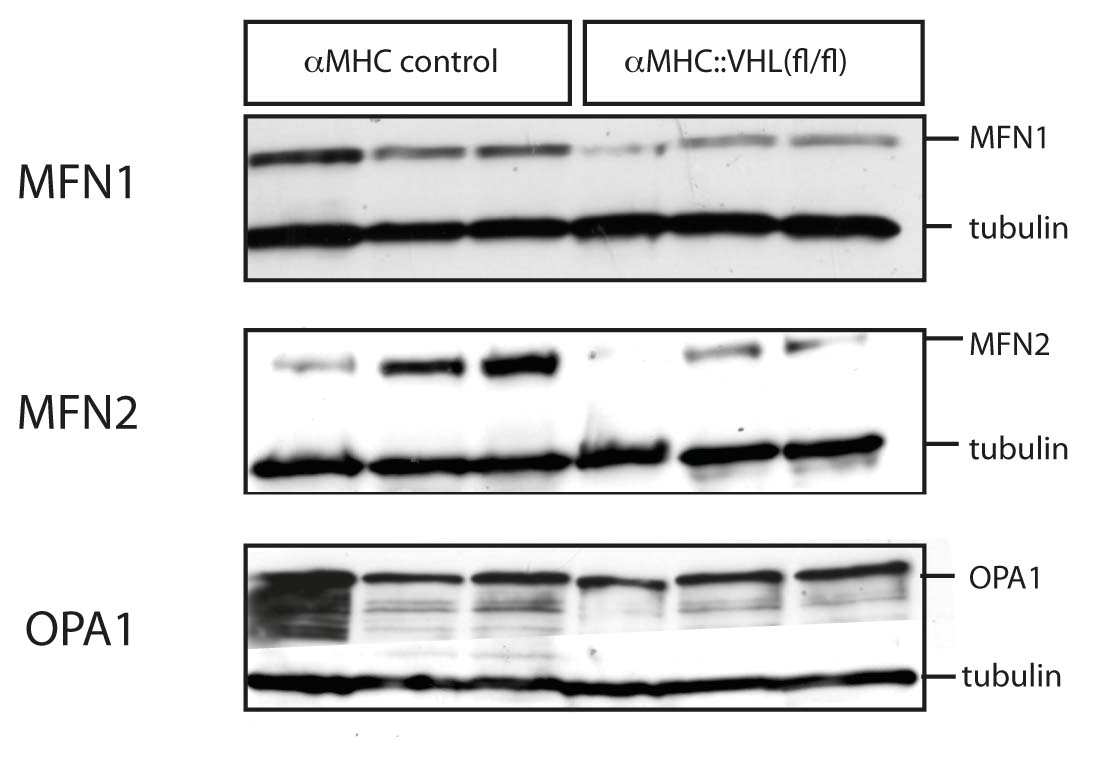


**Supplementary figure 3**

Representative western blots of protein extracts from αMHC::VHL^(fl/fl)^ and αMHC control hearts, for MFN1, MFN2 and OPA1 with respective tubulin loading controls

**Supplementary Figure 4**

Cardiac protein levels of Drp1 and hFIS do not significantly change around birth (n=6 hearts each group).


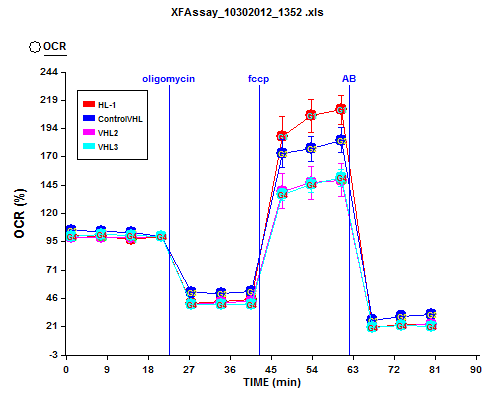


**Supplementary Figure 5**

Seahorse Assay trace for HL1 cells untransfected (HL1), empty vector (ControlVHL) and two independent stably transfected lines expressing a VHL shRNA construct. Error bars represent SD. (OCR; Oxygen consumption rate).


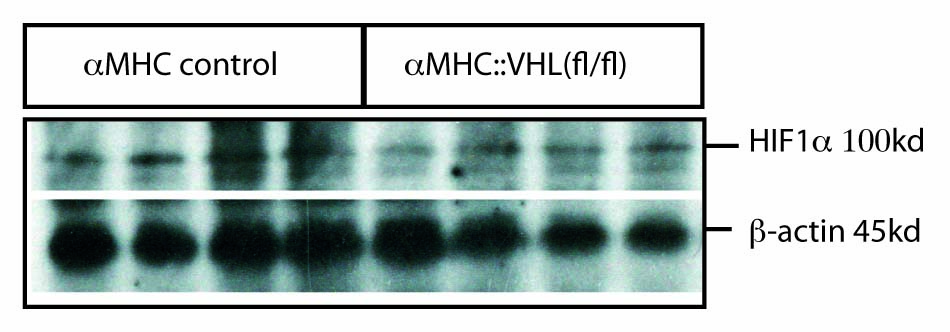


**Supplementary figure 6**

Western blots of protein extract from E18.5 αMHC::VHL^(fl/fl)^ and αMHC control hearts using an anti HIF1α antibody and β actin loading control, showing no significant difference in protein levels of HIF1 α between VHL deleting and control hearts, implying maximal activation of the HIF system *in utero.*

**Supplementary Materials and Methods**

*Embryonic cardiomyocyte isolation*

Embryonic cardiac myocytes were isolated from E16-18 embryos from timed-pregnant mice. The mother was sacrificed by cervical dislocation and embryos were taken and stored at room temperature in freshly prepared ADS buffer (116mM NaCl, 5.4mM KCL, 20mM HEPES, 0.8mM NaH_2_PO_4_, 405.7µM MgSO4, 5.5mM glucose, pH 7.35). Hearts from each embryo were excised and rinsed in ADS buffer before they were stored in labelled 1.5ml Eppendorf tubes. Tails samples were also obtained for PCR analysis. Tissue culture dishes (6 well plates or Seahorse plates) were coated with 1% gelatin (Difco) during the isolation steps.

The following steps were carried out under tissue culture conditions: Hearts were minced and serially digested in 1ml digestion buffer (ABS buffer) containing 1mg/ml Collagenase II (Worthington Chemicals) and 0.5mg/ml Pancreatin (Sigma-Aldrich) in a shaking incubator set at 37°C for 15 minutes. Myocytes were not removed from the first digestion therefore the supernatant from the first digest was discarded without removing the hearts. Fresh digestion buffer was added to each heart sample and left to incubate at 37°C for 10 minutes. After this incubation, the digest was removed and transferred to 15ml falcon tubes containing 2ml Fetal Calf serum (FCS) to stop the enzymic reaction. The supernatant collected from two digests were centrifuged at 1000 r.p.m for 3 minutes, the solution was discarded and the cell pellet resuspended in fresh FCS. These incubation and centrifugation steps were repeated six to eight times until the cardiac myocytes were completely removed and the heart tissue was reduced to a single matrix conglomerate.

The final cell pellet from each heart was resuspended in Dulbecco’s Modified Eagle’s Medium (DMEM) containing 10% FCS and penicillin (50 U/ml), streptomycin (50 µg/ml) (Invitrogen). Fibroblasts were allowed to attach for 30 minutes and the non-adherent (myocyte) fraction were plated into either 6 well dishes containing 22mm glass coverslips (VWR) for mitochondrial function analysis or Seahorse plates for oxygen consumption analysis. After 24 hours, the myocytes formed a monolayer of spontaneously beating cells, the media was removed and replaced with DMEM containing 1% FCS and 1% P/S before further analysis. Hearts from Cre positive or *Vhl* floxed embryos were used as controls to compare against mutant *Vhl* hearts.

**Chromatin Immunoprecipitation Primers**

|  | 5’ | 3’ |
| --- | --- | --- |
| MFN1 |  |  |
| -28730 | CCCAGATGGTTTAAGCCTCTTT | ACCAGATGCACCAAGTTCCTC |
| -28140 | TCTTTGTTCATGCATCACGTGC | TTTATTGGCAGAGGTGGCCTC |
| -27950 | CACAGGAGATAGCAGTTGTCGG | AATTGTGTGAAACCACGCCA |
| -21770 | TCACTGAGTTATTCGCCAGCC | CTCACTGTTGATCTCTAGCGGC |
| -20641 | CACCCCAGTTTGTCAGTTCTCA | AATTCCAATGGCTCGCTCC |
| MFN2 |  |  |
| -8330 | ACAGTTGCCACCAGATGCTGT | CCAATCCAGGTGCATCCAA |
| -7560 | TTCCAGACAAGAAGCTGCCCT | GCCTCAGAGGTAAATCAGCACG |
| -6110 | CATCTCTCTAGGCCCCAACCAT | GGTGGAAGGAAAGAACCTGCTC |
| -2100 | TGAGGATGGCCTTAAGCTCTG | CACAAGTTGTGTGACCTCCACA |
| OPA1 |  |  |
| -4565 | CACGAAACCCTAAGAGCAGGAC | GCAATACAATGAGACAGGCCCT |
| -4145 | GAAAGGCTGCAAGAGCTCAGA | CAATGATTGAACACTGGTGGCT |
| -2325 | GGAGTGTAATGACGATGCGTGA | TGGTATCCAAGGTCCCAGAGGA |
